# Bilateral Three-Port Video-Assisted Thoracoscopic Thymectomy for Thymoma in Good’s Syndrome With a History of Bacteremia

**DOI:** 10.7759/cureus.67380

**Published:** 2024-08-21

**Authors:** Ryo Shibayama, Sakashi Fujimori, Souichirou Suzuki, Reo Ohtsuka, Takahiro Karasaki

**Affiliations:** 1 Gastrointestinal Surgery, Toranomon Hospital, Tokyo, JPN; 2 Respiratory Surgery, Toranomon Hospital, Tokyo, JPN

**Keywords:** perioperative infection control, minimally invasive surgery, good’s syndrome, total thymectomy, three-port vats

## Abstract

A 62-year-old man presented with back pain, lower leg swelling, and fever and was referred to our hospital. Blood cultures identified *Helicobacter fennelliae* as the causative agent of bacteremia associated with pyogenic spondylitis and cellulitis. CT revealed a tumor in the upper anterior mediastinum, and blood tests showed low gamma globulin levels, raising the suspicion of Good’s syndrome. Infection control was prioritized, and the patient received antibiotics for four weeks. After blood cultures returned negative, preoperative gamma globulin was administered to mitigate infection risk, and a total thymectomy was planned. A bilateral three-port thoracoscopic total thymectomy was performed, and the patient was observed as an outpatient without any postoperative infection recurrence. We present a case of Good’s syndrome with a high infection risk, successfully managed with a minimally invasive bilateral three-port thoracoscopic total thymectomy and effective perioperative infection control.

## Introduction

Good’s syndrome, characterized by thymoma associated with hypogammaglobulinemia, was first described by Good in 1954 [1]. It is a rare condition, affecting approximately 0.4-0.6% of patients with thymomas [2,3]. The disease often has a poor prognosis, with severe postsurgical infections being a major cause of mortality and a low 10-year survival rate of 33% [4]. Sinopulmonary infections are the most common, and about 50% of patients experience diarrhea, often with unidentified pathogens [4]. We report a unique case of thymoma with Good’s syndrome in which the patient had a history of bacteremia from pyogenic spondylitis and cellulitis but successfully underwent bilateral three-port thoracoscopic total thymectomy without postoperative infection recurrence. To our knowledge, this case represents a novel report.

## Case presentation

A 62-year-old male patient was referred to our hospital after presenting with back pain, lower leg swelling, and fever. He was initially evaluated by another department within the same institute. Following a thorough examination, pyogenic spondylitis was suspected, and blood cultures yielded positive results for *Helicobacter fennelliae*. The patient was treated with ampicillin (ABPC) for four weeks and then referred to our department to discuss a subsequent surgical procedure (thymectomy) for an anterior mediastinal tumor. Laboratory findings revealed an IgG level of 625 mg/dL (normal range: 870-1,100 mg/dL).

Imaging findings

**Figure 1:**
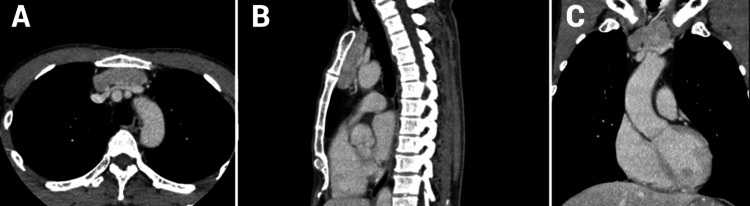
CT imaging of the thoracic aorta (A) The axial cross-section displays the mass in the anterior mediastinum, along with the thoracic aorta and surrounding structures. (B) The sagittal plane illustrates the vertical extent of the mass relative to the spinal column and thoracic aorta. (C) The coronal view shows the mass in relation to the aortic arch and adjacent anatomical features, including its proximity to the internal thoracic arteriovenous veins and the left brachiocephalic vein.

CT revealed an 80 × 57 × 17 mm lobulated mass in the anterior mediastinum, located at the upper margin of the sternal pattern, in bilateral contact with the internal thoracic arteriovenous veins, and posteriorly approaching the left brachiocephalic vein (Figure 1).

**Figure 2:**
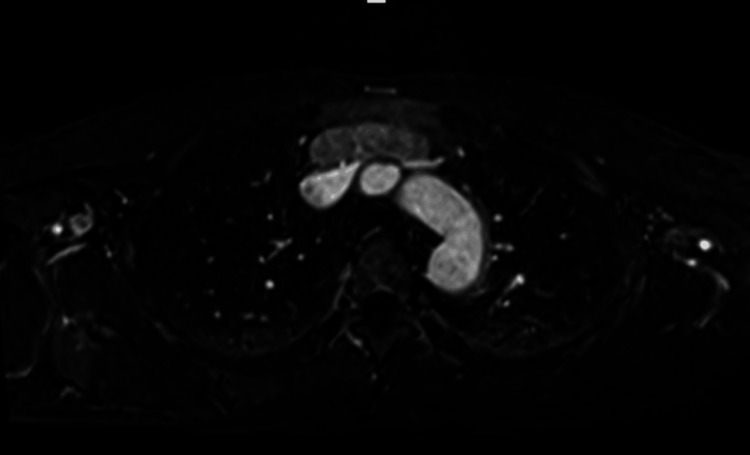
MRI image of the thoracic aorta and mediastinal tumor This T2-weighted MRI scan provides a cross-sectional view of the thoracic aorta and surrounding structures, including a mediastinal tumor. The aorta appears bright white, indicating blood flow, while the mediastinal tumor is visible as a distinct mass, separated from the left brachiocephalic vein by a thin layer. The darker areas represent surrounding tissues and organs within the chest cavity.

Mediastinal MRI revealed a tumor, which was predominantly a distinct entity separated from the left brachiocephalic vein by a single layer (Figure 2).

**Figure 3:**
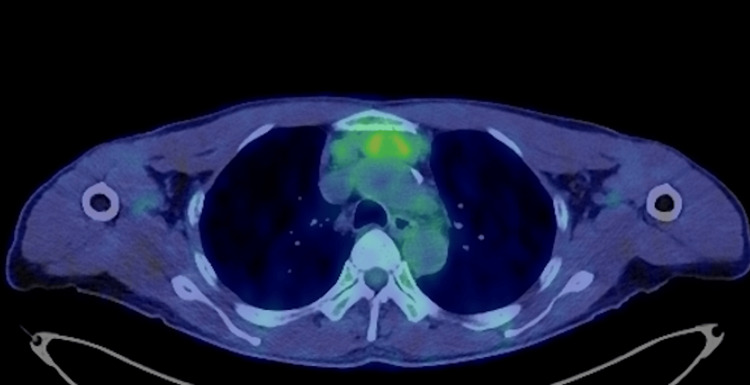
Axial PET/CT image of the thorax showing FDG accumulation This axial PET/CT image of the thorax shows areas of mild abnormal FDG accumulation within the tumor. The SUV max/peak is 3.05/2.51, indicating metabolic activity. Regions with increased FDG uptake are highlighted in green and yellow, suggesting potential pathological conditions such as inflammation or malignancy. FDG, fluorodeoxyglucose; SUV, standardized uptake value

PET/CT revealed mild abnormal fluorodeoxyglucose accumulation in the tumor, with a standardized uptake value max/peak of 3.05/2.51 (Figure 3).

Preoperative course

Although thymectomy may not improve immunodeficiency in Good’s syndrome, an interdisciplinary team determined it to be the appropriate oncologic approach for this case of suspected thymoma. Given the patient’s recent severe infection, surgery was scheduled after obtaining negative blood culture results from samples taken about one week following antibiotic treatment. During the perioperative period, IV immunoglobulin was administered twice (10 g and then 5 g), and ABPC was continued. The patient was planned for a bilateral three-port thoracoscopic total thymectomy. Additionally, a neck incision was prepared in case of poor visualization, and a median sternotomy was planned if tumor invasion of the left brachiocephalic vein required combined resection.

Surgical findings

The surgery began with the patient in the right lateral recumbent position. Three skin incisions were made on the left chest, and three ports were inserted: a 12 mm port at the midaxillary line in the fifth intercostal space, a 5 mm camera port at the posterior axillary line in the fourth intercostal space, and a 7 mm port at the anterior axillary line in the third intercostal space. The mediastinal pleura was opened along the left phrenic nerve, allowing dissection of the tumor from the pericardium and sternum. The left internal thoracic vein was centrally and peripherally ligated and dissected to visualize the space between the left subclavian vein and the chest wall. Although the tumor’s upper edge was at the upper margin of the sternum, it was dissected circumferentially and retracted downward by dividing the inferior thyroid vein and other vessels between the chest wall and the subclavian vein. Since the tumor did not invade the left brachiocephalic vein, a cervical incision or median sternotomy was deemed unnecessary.

The patient was then repositioned to the left lateral recumbent position. Three skin incisions were made on the right chest, and three ports were inserted: a 12 mm port at the midaxillary line in the fifth intercostal space, a 5 mm camera port at the anterior axillary line in the fourth intercostal space, and a 7 mm port at the posterior axillary line in the third intercostal space. The mediastinal pleura was opened along the right phrenic nerve, and the dissected surfaces were brought into contact with the contralateral dissected surfaces to complete the total thymectomy.

Pathological findings

**Figure 4:**
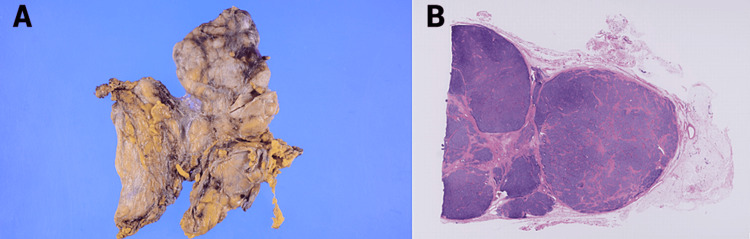
Gross and histopathological examination of type AB thymoma (A) This image displays the gross morphology of an 80 × 60 × 18 mm lobulated thymoma covered with a thin capsule, classified as Stage II according to the Masaoka classification. (B) This image shows a histopathological section of the tumor, stained with H&E (×10), identified as a type AB thymoma per the World Health Organization classification. The section confirms no tumor exposure at the resection margin, the presence of lymphatic invasion, and the absence of venous invasion and lymph node metastasis.

An 80 × 60 × 18 mm lobulated tumor covered with a thin capsule was observed. According to the Masaoka classification, the tumor was Stage II. Histologically, it was identified as a type AB thymoma based on the World Health Organization histopathological classification. There was no tumor exposure at the resection margin. The tumor showed lymphatic invasion but no venous invasion or lymph node metastasis (Figure 4).

Postoperative course

The patient was discharged on postoperative day four and has been undergoing monthly outpatient follow-ups. One year after the surgery, there was no recurrence of infection; however, IgG levels remained low at 580 mg/dL. Based on the pathological diagnosis, the patient is being monitored without additional treatment for thymoma [5].

## Discussion

A search for Good’s syndrome, surgery, or thymectomy in Japanese medical journals and PubMed up to December 2023 revealed 38 reports in Japan. Table 1 summarizes these 39 cases, including the current one. Few reports address surgery for Good’s syndrome, particularly considering the high risk of postoperative infection due to preexisting infections.

**Table 1:** Comparison of clinical characteristics and surgical outcomes in patients diagnosed with Good’s syndrome VATS, video-assisted thoracoscopic surgery

Characteristics and classifications	Total	Diagnosed with Good’s syndrome	
		Preoperatively (n = 25)	Postoperatively (n = 14)
Sex			
Male	24	17	7
Female	15	8	7
Age (years, mean ± SD)	60.8 ± 11.09	58.7 ± 10.74	64.5 ± 10.74
From surgery to diagnosis (month, mean ± SD)	-	-	36.3 ± 45.28 (5 days to 13 years)
Coexistence with infection	27	13	14
Without infection	12	12	0
WHO classification			
A	2	1	1
AB	12	7	5
B1	3	2	0
B2	6	2	4
B3	2	2	0
Unknown	15	11	4
Masaoka classification			
I	12	6	6
II	1	1	0
III	3	2	1
IVb	1	1	0
Unknown	22	15	7
Tumor diameter (mm, mean ± SD)	65.3 ± 24.03 (n = 15)	63.7 ± 24.70 (n = 11)	70 ± 21.21 (n = 4)
Approach of surgery			
Median sternotomy	22	14	8
VATS	6	4	2
Unknown	11	7	4

Surgery and adjuvant therapy are considered for managing Good’s syndrome, especially for treating thymomas. However, there are no Japanese reports indicating postoperative improvement in gamma globulin levels. The primary aim of surgery is to address thymomas, which, while often displaying indolent spindle cells, can occasionally be malignant [6]. Hypogammaglobulinemia is a notable prognostic factor. Reports have highlighted deaths related to postoperative pneumonia, infection of the midline sternotomy wound, and mediastinitis [7].

The timing and safety of surgery are crucial for infection control, given the potential for coexisting infections. There is no established consensus on perioperative infection control, and periodic postoperative replacement therapy is recommended [8]. In cases of poor infection control, conservative treatment is preferred for Good’s syndrome.

Future implications

Accumulating more cases is crucial for standardizing safe surgical techniques for patients with Good’s syndrome complicated by serious infections.

## Conclusions

We performed a total thymectomy using a bilateral three-port thoracoscopic approach. This method was chosen because it allowed for the dissection of the internal thoracic vessels, provided a clear view between the chest wall and subclavian vein, and enabled the tumor to be separated from the left brachiocephalic vein. Most reports describe thymectomy through median sternotomy; however, there have been no documented cases of serious infections, such as bacteremia, as seen in the present case. Effective perioperative infection control and the use of minimally invasive surgery can enhance safety for high-risk patients.
